# Co-ocurrence of the idiopathic scoliosis and the malocclusion– early results

**DOI:** 10.1186/1748-7161-7-S1-O67

**Published:** 2012-01-27

**Authors:** M Tyrakowski, M Laskowska, J Czubak, D Olczak-Kowalczyk

**Affiliations:** 1 Departament of Orthopaedics, Pediatric Orthopaedics and Traumatology Postgraduate Medical Education Centre in Warsaw, Otwock, Poland; 2 Orthodontic Department, Medical University of Warsaw, Poland; 3 Department of Pediatric Dentistry, Medical University of Warsaw, Poland

## Backgroud

Proper timing of the treatment of idiopathic scoliosis seems to be crucial. That is why we tried to look for other abnormalities that may occur more often in children with scoliosis and thus enable earlier establishing the diagnosis of scoliosis.

The aim of the study was to evaluate the co-occurence of idiopathic scoliosis and malocclusion [1-4].

## Materials and methods

Material consisted of 52 consecutive patients with idiopathic scoliosis that visited The Department of Pediatric Orthopaedics (out-patients and hospitalized) between February 1st and December 31st 2010. The mean age of the patients was 14 years (8,4 – 18,9). There were 48 girls and 4 boys. All the patients were examined by an orthopaedic surgeon (trunk rotation measured by use of Bunnell’s scoliometer). The diagnosis of scoliosis was based on X-rays. All the patients with non-idiopathic scoliosis were excluded from the research. Next the patients with scoliosis were examined by orthodontic specialist (physical examination and photos).

## Results

51 patients had the malocclusion and only one patient was found with a correct occlusion. There were 23 cases of posterior occlusion, 9 - partially lateral cross bite, and 19 with other anomalies. Among the 52 examined patients only 18 had undergone previous orthodontic treatment.

## Conclusions

We present the early results. However, we found higher incidence of malocclusion in the scoliotic patients than in general population. Our results may suggest that all the children and adolescents treated because of malocclusion should be examined by an orthopeadic surgeon. On the other hand scoliotic patients may need orthodontic treatment.
